# Study protocol of a prospective multicenter study comparing (cost-)effectiveness of a tailored interdisciplinary head and neck rehabilitation program to usual supportive care for patients treated with concomitant chemo- or bioradiotherapy

**DOI:** 10.1186/s12885-019-5874-z

**Published:** 2019-07-03

**Authors:** Ann-Jean C. C. Beck, Ellen Passchier, Valesca P. Retèl, Martijn M. Stuiver, Lisette van der Molen, Willem M. C. Klop, Arash Navran, Wim H. van Harten, Michiel W. M. van den Brekel

**Affiliations:** 1 Department of Head and Neck Surgery and Oncology, the Netherlands Cancer Institute, Plesmanlaan 121, 1066 CX Amsterdam, the Netherlands; 2 Division of Psychosocial Research and Epidemiology, the Netherlands Cancer Institute, Amsterdam, the Netherlands; 3 Center for Quality of Life, Netherlands Cancer Institute, Amsterdam, the Netherlands; 4 ACHIEVE Center of Applied Research, Faculty of Health, Amsterdam University of Applied Sciences, Amsterdam, the Netherlands; 5 Department of Health Technology and Services Research, University of Twente, Enschede, the Netherlands; 6 Rijnstate Hospital, Arnhem, the Netherlands; 7 Department of Radiation Oncology, Netherlands Cancer Institute, Amsterdam, the Netherlands; 8 Institute of Phonetic Sciences, University of Amsterdam, Amsterdam, the Netherlands; 9 Department of Oral and Maxillofacial Surgery, Amsterdam University Medical Center (Amsterdam UMC), Amsterdam, the Netherlands

**Keywords:** Rehabilitation, Interdisciplinary care, Multidisciplinary care, Head and neck cancer, (cost-) effectiveness, Quality of life, Societal participation, Return to work

## Abstract

**Background:**

Since 2011, a tailored, interdisciplinary head and neck rehabilitation (IHNR) program, covered by the basic healthcare insurance, is offered to advanced head and neck cancer (HNC) patients in the Netherlands Cancer Institute (NKI). This program is developed to preserve or restore patients’ functioning, and to optimize health-related quality of life (HRQoL). It applies an integrated approach to define patients’ individual goals and provide rehabilitation care throughout the cancer care continuum. The aim of the current study is to assess the (cost-) effectiveness of the IHNR approach compared to usual supportive care (USC) consisting of monodisciplinary and multidisciplinary care in advanced HNC patients.

**Methods:**

This multicenter prospective observational study is designed to compare (cost-)effectiveness of the IHNR to USC for advanced HNC patients treated with chemoradiotherapy (CRT) or bioradiotherapy (BRT). The primary outcome is HRQoL represented in the EORTC QLQ-C30 summary score. Functional HRQoL, societal participation, utility values, return to work (RTW), unmet needs (UN), patient satisfaction and clinical outcomes are secondary outcomes, assessed using the EORTC QLQ-H&N35, USER-P, EQ-5D-5 L, and study-specific questionnaires, respectively. Both patient groups (required sample size: 64 per arm) are requested to complete the questionnaires at: diagnosis (baseline; T0), 3 months (T1), 6 months (T2), 9 months (T3) and 12 months (T4) after start of medical treatment. Differences in outcomes between the intervention and control group will be analyzed using mixed effects models, Chi-square test and descriptive statistics. In addition, a cost-effectiveness analysis (CEA) will be performed by means of a Markov decision model. The CEA will be performed using a societal perspective of the Netherlands.

**Discussion:**

This prospective multicenter study will provide evidence on the effectiveness and cost-effectiveness of IHNR compared to USC. RTW and societal participation, included as secondary outcomes, have not been studied sufficiently yet in cancer rehabilitation. Interdisciplinary rehabilitation has not yet been implemented as usual care in all centers, which offers the opportunity to perform a controlled clinical study. If demonstrated to be (cost-)effective, national provision of the program can probably be advised.

**Trial registration:**

The study has been retrospectively registered in the Netherlands Trial Registry on April 24th 2018 (NTR7140).

## Background

In the Netherlands, approximately 3200 patients are diagnosed with head and neck cancer (HNC) annually [1]. Cancer of the head and neck is often treated curatively by (a combination of) surgery, radiotherapy and/or chemotherapy. As a consequence of the tumor and its treatment, impairment of functioning may occur concerning e.g. swallowing, speech, breathing and cancer-related fatigue, but also psychosocial problems such as altered body image, anxiety and depression. Additionally, patients may suffer from pre-existing comorbidity relating to physical and/or psychosocial functioning. Rehabilitation care can play an important role in restoring these functions, and may help to regain daily life activities and improve health-related quality of life (HRQoL) [2, 3].

Rehabilitation often comprises monodisciplinary interventions, also known in the Netherlands as ‘usual supportive care’ (USC), provided by specialized individual healthcare professionals. Nonetheless, monodisciplinary care does not always sufficiently meet patients’ needs, as problems are often multifactorial and complex [2]. To optimize the rehabilitation of patients, an upcoming trend is to implement multidisciplinary rehabilitation care, the importance of which is underlined in the guideline on cancer rehabilitation developed by the Netherlands Comprehensive Cancer Organization (*Integraal Kankercentrum Nederland* - IKNL) [4]. The rationale is that a coordinated multidisciplinary approach, in which healthcare professionals cooperate to optimize patients’ outcomes, might be more effective than healthcare professionals individually addressing patients’ problems during conventional monodisciplinary rehabilitation care. In multidisciplinary care, different healthcare professionals have separate (sub)goals that are achieved during rehabilitation with the patient. When these goals are aligned with the objective to achieve one broader goal, such as regaining participation in society by the patient, this is defined as ‘interdisciplinary care’. This type of care is assumed to be especially useful when patients have several interrelated and/or severe problems, which is often the case in advanced HNC [5–12]. However, it is also recognized that this type of rehabilitation can be more expensive.

The integrative, biopsychosocial, International Classification of Functioning, Disability and Health (ICF) model [7, 13], developed by the World Health Organization, is often applied as a framework for interdisciplinary rehabilitation. The ICF model describes individual functioning in a broader context, consisting of two parts: (1) Functioning and Disability and (2) Contextual factors. Functioning and Disability encompasses the physical and functional status; Contextual factors are subdivided in environmental and personal factors (e.g. coping strategies). In addition, a distinction is made between capacity (the ability to execute a task or action) and performance (the actual task or activity performed in daily life). Discrepancies in current and desired status in each of these components determine a person’s individual rehabilitation objective to be achieved, and consequently, the interdisciplinary interventions to apply. For example, a male HNC patient treated with chemoradiotherapy (CRT), who cannot perform daily activities due to feeding tube dependency and fatigue. The activities (e.g. eating and drinking, walking and driving) this person wants to do, relate to the individual roles in his daily life (e.g. being a father, working as a bus driver). Both components determine which tailored interventions to apply. For example, to be able to perform daily activities such as eating and drinking, walking and driving, swallowing rehabilitation and physical exercise will be needed respectively, both combined with nutritional advice for a personalized, balanced diet. These interventions aim to optimize the patient’s capacity. Besides optimizing the patient’s capacity, especially if functional improvement is limited, rehabilitation goals can be achieved also by addressing behavioral and/or environmental factors. To optimize the patient’s performance in order to resume his role as father and as bus driver, interventions such as energy coaching and family counseling could be applied. These interventions will address personal factors, such as coping, and will use cognitive behavioral therapy to improve the ability to adjust to limitations and improve social functioning. As both physical- and cognitive-based interventions are executed simultaneously within interdisciplinary rehabilitation care, this approach can have a synergistic effect. For HNC patients, a specific ICF HNC core set is available to facilitate interdisciplinary communication within rehabilitation [14].

A HNC-specific interdisciplinary rehabilitation program (IHNR) was developed in the Netherlands Cancer Institute (NKI) in 2010 (version 1.0), based on the ICF framework. IHNR consists of structured interdisciplinary interventions, tailored to the individual needs of the patient, with the primary aim to enable patients to regain their desired level of participation in society. This program is integrated into medical care, which means that the rehabilitation care is offered throughout cancer treatment. IHNR is a modular program (including swallowing rehabilitation module, eating module, bodyweight monitoring module, preventive shoulder rehabilitation module, physical exercise module, energy conservation module, guidance coping and adjustment module, art therapy module). Each module is based on the best available evidence. Healthcare professionals that can be consulted within IHNR, apart from the head and neck surgeon, radiotherapist, physical medicine and rehabilitation (PM&R) physician and dentist, are: the speech-language pathologist, dietician, physical therapist, occupational therapist, medical social worker and/or psychologist, and art therapist [15]. IHNR is implemented as standard care in the rehabilitation of HNC patients in the NKI. More details on this program are given in the Methods section.

The program was found feasible in a previous observational study. In this study, positive outcomes on HRQoL were observed in patients who participated with the IHNR compared to reference values [16]. Also, the time until recovery was shorter than usually observed for patients treated with USC (estimated approximately 1 year) [2]. In addition, the preventive (swallowing) exercise program (PREP) included in the IHNR, was found cost-effective compared to USC in advanced HNC patients treated with CRT [17]. So far, there is limited uptake of this program by other HNC care providers, partly because of the character of the evidence, partly because insurance agencies for the same reason often do not want to engage in contracting additional services for this population.

The added value of interdisciplinary and multidisciplinary cancer rehabilitation compared to monodisciplinary care, in terms of effectiveness and cost-effectiveness, are reported scarcely in literature for cancer patients [18]. In addition, the effect of this integrated IHNR program on HRQoL, return to work (RTW), participation in society and cost-effectiveness compared to USC has not been studied previously in a controlled setting.

Therefore, the aim of our study is to investigate the effectiveness and cost-effectiveness of IHNR (intervention group) compared to USC (control group) in advanced HNC patients treated with concomitant CRT or bioradiotherapy (BRT) in a prospective controlled clinical study.

Prior to this study, we framed three hypotheses. First, we hypothesize that IHNR will shorten the time to regain (baseline) HRQoL [2]. Second, we hypothesize that the program will enhance the ability to resume work-related and daily activities, and will lead to a reduction in medical consumption (e.g. tube feeding) and adverse events (e.g. occurrence of pneumonias). Third, we expect that these improvements will result in a reduction of hospital- and society-related costs, resulting in a more cost-effective approach than USC [17].

## Methods

### Study design

We will perform a prospective controlled observational study comparing the effectiveness and cost-effectiveness of IHNR to USC for advanced HNC patients using Patient Reported Outcome Measures (PROMs). Primary objective is HRQoL. Secondary outcomes are functional HRQoL, return to work, societal participation, cost-effectiveness, unmet needs, clinical outcomes and patient satisfaction. Before the start of the treatment, patients in the intervention group are offered to participate in the IHNR. The intervention group consists of all eligible consenting patients treated in the NKI, despite participating or not in the program. The control group consists of advanced HNC patients treated in six Dutch HNC centers which are representative for the USC in the Netherlands; three academic and three community centers, providing mono- or multidisciplinary care.

This study does not fall under the Medical Research Involving Human Subjects Act (*Wet Medisch Wetenschappelijk Onderzoek*) due to the non-invasive nature of the study, but is submitted to and approved by the Dutch Medical Ethical Committees (registered: P16HNR). The study started in February 2017.

### Study population: in- and exclusion criteria

Adult patients diagnosed with advanced head and neck squamous cell carcinoma (HNSCC; stage 3 and 4) are included in this study. Patients are eligible if they are to be treated with primary CRT (Cisplatin or Carboplatin) or BRT (Cetuximab) with intent to cure. IHNR takes place mainly at the Center for Quality of Life in the outpatient clinic of the NKI. Patients who are unwilling to cooperate in the study or unable to take part in the program due to a language barrier or an interfering psychiatric or psychological disorder are excluded from the study. Advanced HNC patients who are treated primarily with surgery are not eligible for the study, in order to control heterogeneity within the two arms and ensure comparability between the arms. At least 64 patients are needed per arm.

### Study groups

#### IHNR – intervention group

Since 2011, IHNR is offered to HNC patients as standard rehabilitation care in the NKI, and it is reimbursed through the basic health care insurance package. Recently, the program has been updated to the newest scientific literature and clinical experience (HNR version 2.0, 2016) [15].

IHNR begins after diagnosis prior to or at the start of oncological treatment and continues until approximately 6 months post treatment [2]. The PM&R physician defines in discussion with the patient relevant rehabilitation needs and goals, and the core problem that needs to be addressed during rehabilitation. Subsequently, the PM&R physician determines which treatment modules can be applied during treatment. Preventive swallowing rehabilitation combined with nutritional assessment and advice is routinely offered during CRT and BRT. Other interventions are initiated as deemed appropriate to achieve the intended and defined goals, and include physical exercise supervised by a physical therapist, energy counseling or RTW guidance by an occupational therapist, and psychosocial care by a medical social worker and/or psychologist, and art therapist. In conversation with the patient, expected length and frequency of the rehabilitation interventions and the various healthcare professionals to be involved are clarified. Thereafter, the PM&R physician refers to relevant healthcare professionals depending on the rehabilitation modules selected. Assessments are made before the start of rehabilitation treatment by each involved health professional. At the end of the intake phase, the patient’s core problem and individual rehabilitation needs, as well as the results of the assessments are discussed in an interdisciplinary team meeting. Subsequently, several SMART (Specific, Measurable, Attainable, Realistic, Time-bound) interdisciplinary rehabilitation (sub)goals are formulated. During IHNR, tailored interventions are offered to the patient that meet the individual goals. The interventions are provided individually, or in group sessions if applicable and indicated.

All goals are evaluated every 4 to 6 weeks within the rehabilitation team. Besides the PM&R physician and healthcare professionals, a head and neck surgeon and radiotherapist attend the rehabilitation interdisciplinary meetings to discuss interference of the oncological treatment, and its consequences for the individual rehabilitation plan. The dentist and oral hygienist can be involved as well. This integrated approach distinguishes IHNR from other rehabilitation programs [2].

#### USC – control group

The control group comprises 6 hospitals, all of which are members of the Dutch Head and Neck Society (DHNS). USC is mostly delivered by healthcare professionals who are affiliated with the Dutch working group of allied healthcare in HNC (PWHHT), and follow national guidelines for HNC supportive care [19]. Nevertheless, from practice, we know that the content and organizational structure between centers can vary between these national centers.

In one subpopulation of the control group, an academic center, HNC patients are offered multidisciplinary rehabilitation care 6 to 8 weeks after treatment. A personalized approach starts from the third chemotherapy cycle with monitoring by the speech-language pathologist and the dietician to offer advice when compensation is needed to guarantee safe and sufficient intake of liquid and food. Patients who become dependent on non-oral intake receive individual coaching to keep drinking sips of water regularly, despite pain. At 6 to 8 weeks after completion of the oncological treatment, the patient’s condition is evaluated by a multidisciplinary team and when needed the patient is assessed by a PM&R physician, dietician, occupational therapist, physical therapist and speech-language pathologist, usually resulting in a rehabilitation plan. This subgroup is however reflected as usual care because it rather reflects common practice as patients are included after treatment and there is no structured interdisciplinary care present during rehabilitation.

In general, the other centers in the control group offer monodisciplinary rehabilitation care on indication during or after treatment. The disciplines involved during rehabilitation differ among the centers.

### Recruitment and completion of PROMs

**Fig. 1:**
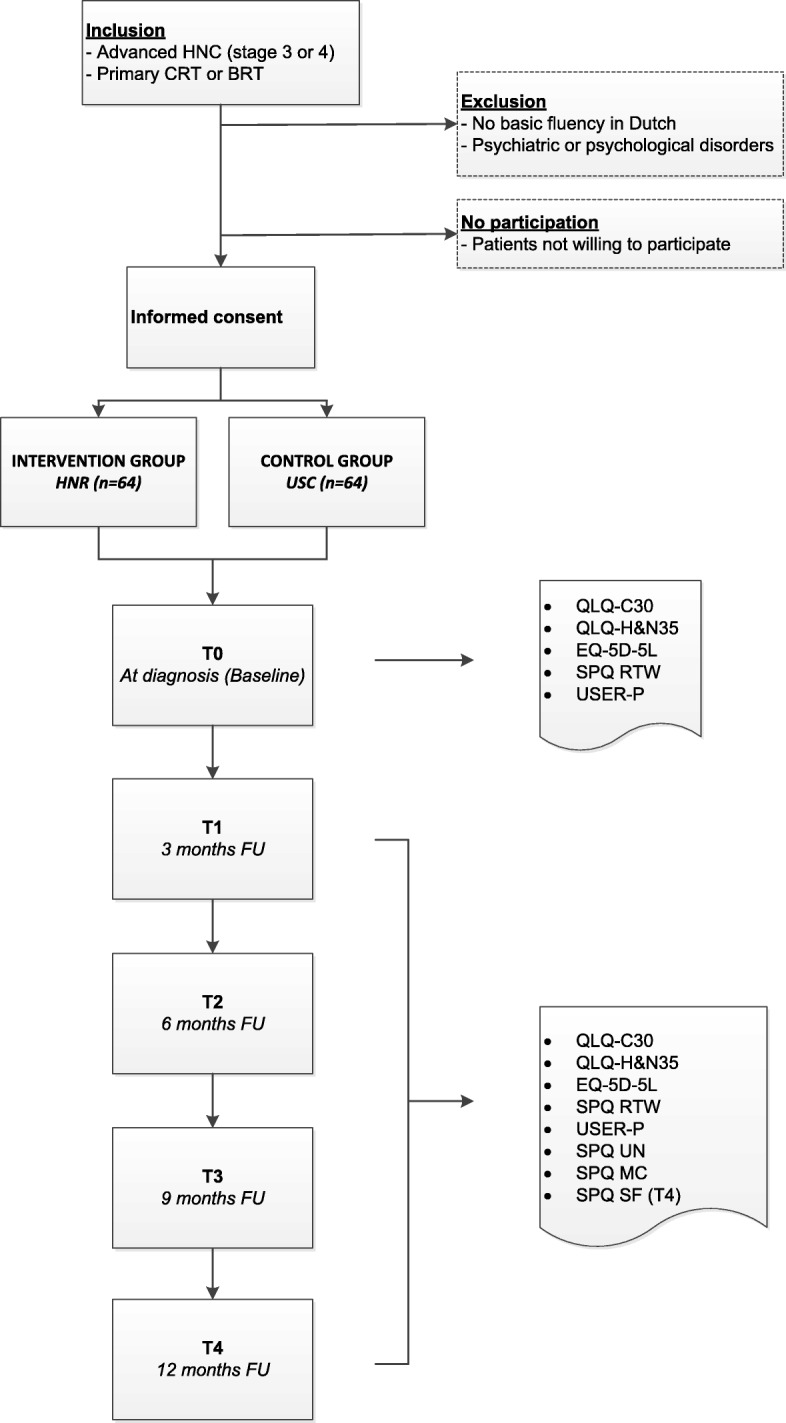
Flowchart of the study Abbreviations: BRT, bioradiotherapy; CRT, chemoradiotherapy; EQ-5D-5 L, five-level EuroQol five-dimensional questionnaire; FU, follow-up; HNC, head and neck cancer; IHNR, interdisciplinary head and neck cancer rehabilitation program; MC, medical consumption; QLQ-C30, Quality of Life Questionnaire-Core30; QLQ-H&N35, Quality of Life Questionnaire-Head and Neck35; RTW, return to work; SF, satisfaction; SPQ, study-specific questionnaire; UN, unmet needs; USC, usual supportive care; USER-P, Utrecht Scale for Evaluation of Rehabilitation-Participation

Patients’ eligibility is assessed at the outpatient clinic by a healthcare professional at the department of the Head and Neck Surgery and Oncology, usually the head and neck surgeon or nurse practitioner. Eligible patients are informed about the study by the investigators of the study or a contact person in the respective centers, usually a healthcare professional of the rehabilitation team. Patient information, informed consent (patient and hospital copy) and a baseline (T0) questionnaire are handed to the patient at the outpatient clinic. Eligible patients who are willing to participate return written informed consent (hospital copy) and the completed baseline questionnaire to the outpatient clinic or by mail. The questionnaire comprises five PROMs concerning HRQoL, societal participation, employment status, medical consumption, unmet needs and patient satisfaction. Follow-up (FU) questionnaires are send to the home address on paper on four different time points during FU within a one-year range: 3 (T1), 6 (T2), 9 (T3) and 12 (T4) months after start of treatment (Fig. 1).

### Primary outcome

#### Effectiveness: quality of life

Primary outcome is assessed at all time points (T0 to T4), and consists of the summary score of the European Organization for Research and Treatment of Cancer Quality of Life Questionnaire-Core 30 (EORTC QLQ-C30) [20].

The EORTC QLQ-C30 comprises 30 questions, that relate to one global health status/QoL scale, five functional scales (physical functioning, role functioning, emotional functioning, cognitive functioning and social functioning), three symptom scales (fatigue, nausea/vomiting and pain) and six single-item scales (dyspnea, insomnia, appetite loss, constipation, diarrhea and financial difficulties). In the EORTC QLQ-C30, each scale or item results in a score ranging from 0 to 100. An increasing score derived from functional scales indicates improved functioning, whereas an increase in symptom scores indicates worsening of symptoms [21, 22]. The EORTC QLQ- C30 summary score originates from all scales except for the global health status/QoL and financial difficulties scales. The score consists of an outcome between 0 and 100 and reflects the overall HRQoL [20].

### Secondary outcomes

Secondary outcomes are assessed at all time points (T0 to T4), except for medical consumption, unmet needs and patient satisfaction with care. Information on medical consumption and unmet needs are obtained from T1 to T4; satisfaction by the patient will be assessed at T4.

#### Head and neck cancer-specific quality of life

HNC-specific HRQoL is assessed using the EORTC QLQ module for HNC; the EORTC QLQ-H&N35. This module contains seven symptom scales (pain, swallowing, senses problems, speech problems, trouble with social eating and social contact, and less sexuality) and eleven single-item scales (teeth, opening mouth, dry mouth, sticky saliva, coughing, felt ill, pain killers, nutritional supplements, feeding tube, weight loss, weight gain), resulting in eighteen scores, ranging from 0 to 100, with higher scores indicating higher symptom burden [22, 23].

#### Cost-effectiveness: costs, life years and utilities

We will investigate the cost-effectiveness of IHNR versus USC from a societal perspective. We will determine life years, quality-adjusted life years (QALYs) and costs. Data on life years related to the survival of HNC patients will be sourced from the Netherlands Cancer Registry. QALYs are calculated by multiplying the life years with the utilities. A utility is a score that ranges from 0 to 1, derived from the five-level EuroQol five-dimensional questionnaire (EQ-5D-5 L), a preference-based instrument. The EQ-5D-5 L consists of five dimensions: mobility, self-care, usual activities, pain/discomfort and anxiety/depression [24].

Direct and indirect costs will be included in the analysis. Costs related to healthcare services by healthcare professionals (e.g. physical therapy, nutritional advice, swallowing rehabilitation), medication use (e.g. painkillers, antibiotics) and dietary supplements (including feeding tube dependency). Direct costs for the intervention will be determined by means of the activity based costing (ABC) method [25]. In addition, work-related costs, such as production loss, costs related to primary care and domestic care will be taken into account. The concise version of the Dutch Medical Consumption Questionnaire (MCQ) will be combined with survival data derived from literature. The MCQ informs on the type and number of consultations by healthcare professionals in the primary and secondary care, domestic care, medication use and dietary supplements [26]. In this way, we can also check for potential crossover contamination between the two groups. To estimate the costs, the cost manual for economic evaluations and the overview of Dutch tariffs defined by the Dutch Healthcare Authority (NZa) are consulted [27, 28].

#### Return to work (RTW)

At baseline and FU, two study-specific questions regarding employment status (e.g. full time or part-time employee, self-employed, retired), adapted to the Dutch work-related legislation, and profession are included. In addition, the first item of the workability index (WAI) will be assessed. The WAI first item is an estimation of the individual employee of his or her work capacity on a scale from 0 to 10 (0 indicates that the patient is not capable of working and 10 indicates most optimal work capacity). This first item is commonly applied as an indicator of workability in previous studies. The outcome of the WAI has proved to be a good predictor of a person’s employability [29, 30].

#### Societal participation

The Dutch Utrecht Scale for Evaluation of Rehabilitation-Participation (USER-P) questionnaire will be used to assess societal participation. It contains questions about daily activities and satisfaction with the way in which patients can perform daily activities. The USER-P is a validated questionnaire, and the most commonly used PROM in rehabilitation care in the Netherlands. It comprises 32 items in three scales: frequency, restrictions and satisfaction. Items are accompanied by a five-optional Likert scale. With the algorithm, an average score is calculated between 0 and 100 for each scale. A higher score indicates a better level of societal participation [31].

#### Unmet needs of the patient

A study-specific question is included at T1 to T4 to identify whether there were important needs that remained unaddressed during the rehabilitation care, and if so, which healthcare professionals the patient wished to be involved. It comprises of a yes-no question to ask whether there were needs not addressed during the last 3 months. If yes, patients can appoint the healthcare provider involved in the need. At the end of the program, the distress thermometer and problem list (completion at start and end of treatment) will be discussed with the patient. In addition, during the multidisciplinary team meeting problems are identified which were not properly addressed at the various time points [32–34].

#### Patient satisfaction

Level of satisfaction concerning the IHNR or USC will be assessed using a five-point scale, with 0 indicating very unsatisfied and 5 is very satisfied.

#### Clinical outcomes

Clinical outcomes include adverse events (e.g. pneumonia) during and after treatment, hospital admissions and medication use. These data will be obtained from medical records and a study-specific concise version of the Medical Consumption Questionnaire (MCQ) [26].

### Patient, tumor and treatment characteristics

Sociodemographic data (age, sex, marital status, educational background and employment status) of patients are gathered at baseline. Date of start of medical treatment is used to determine FU time points. Additional clinical data comprising treatment details of CRT and BRT (e.g. dose of systemic treatment, number of systemic cycles intended and provided) and tumor characteristics will be obtained from the medical record system. Information on progression of disease and recurrences will be evaluated throughout the study.

### Power calculation

To estimate the sample size required for this study, we used a one sample t-test power calculation. The power calculation was based on a comparison between the intervention and control group at end of follow-up, using a power (β) of 0.8 and a significance level of 0.05. We will recruit until we have included 128 patients in total for this analysis (64 are needed per arm) to be able to detect the expected effect-size (Cohen’s d) of 0.5 [35]. In our study we will use a repeated-measures design to allow for a more definitive evaluation of within-subject changes in the HRQOL summary score over time. Although, repeated measures can increase statistical power, we opted for a more conservative approach to sample size calculation by assuming a cross-sectional design. This should cover potential design effects such as attrition, or differences in baseline characteristics. Recruitment time is estimated at 2 years.

### Statistical analysis

Scores on the HRQoL questionnaires and the USER-P will be calculated according to published scoring algorithms [22, 24, 31].

We will look at group differences in HRQOL using a mixed effect growth model with random intercept and slope, nested within site (clusters of different hospitals). This approach takes into account the within and between person variability, and deals adequately with missing data [36]. If baseline differences are identified, these variables will be accounted for in the model. In case of non-ignorable dropout, which will be evaluated halfway during the study, we will correct the model for different patterns of missing values [37]. All analyses will be performed on an ‘intention to treat’ basis and will be adjusted for case mix by means of a propensity score analysis. Additional explorative analyses will be done on a ‘per protocol’ basis.

A generalized mixed-effects model using a logistic link function will be used to estimate the effects of IHNR on the proportion of patients at work, compared to USC, at each time point [38]. In this analysis, only patients are included who either are an employee, are self-employed, or do voluntary work at the baseline measurement.

Employment status, unmet needs and satisfaction of the patient will be analysed using descriptive statistics. Group differences in evaluation of satisfaction will be tested by means of the Chi-square test for trend. The unmet needs and the satisfaction of the patient will be evaluated cross-sectional, at each time point and at T4 respectively.

### Cost-effectiveness analysis

The cost-effectiveness of IHNR compared to USC will be assessed using a Markov model including three health states (disease free survival, progression of disease, death (death due to the HNC or other cause)), a three-month cycle duration and a time horizon of 1 year. One year was chosen because patients are likely to recover within 1 year [2]. Production losses will be analyzed by means of the friction cost method [39, 40]. The friction cost method calculates the costs over the friction period; the period in which the patient has not yet been replaced at work by another employee.

The incremental costs-effectiveness ratio (ICER) is calculated by dividing the difference in total costs of IHNR and USC by the difference in QALYs, and indicates the additional costs of IHNR per QALY gained. The mean together with the degree of uncertainty, represented in confidence intervals of the input parameters, will be estimated, and probabilistic sensitivity analyses will be carried out. Visualization of data will be realized by means of a cost-effectiveness plane and cost-effectiveness acceptability curve [41, 42]. A ceiling ratio of €20.000/QALY, corresponding with the Dutch threshold for preventive care, will be used in this analysis [43].

## Discussion

To our knowledge, this is the first prospective multicenter study to evaluate the added value of the integrated character of a HNC interdisciplinary rehabilitation program. The study takes into account important outcomes of rehabilitation, including RTW and societal participation, which have not been sufficiently studied to date. As IHNR is an integrated program which is tailored to patients’ needs by individual and comprehensive assessment, we assume unmet needs are better addressed within this program.

The primary outcome expressed by the EORTC QLQ-C30 summary score, derived from the EORTC QLQ-C30 measurement instrument, offers a more reliable endpoint than the two-item overall QLQ-C30 score, often used in studies [20]. This study will take into account the variations in the provision of rehabilitation care between centers in the control group, due to the multicenter nature of this study including both academic and non-academic hospitals throughout the Netherlands. In addition, the cost-effectiveness analysis included in the study may provide valuable information to support decision-making concerning reimbursement of cancer rehabilitation programs in the Netherlands.

However, several limitations to the study need to be taken into account. Randomization in the current study design was considered not feasible as IHNR is provided as reimbursed standard care in the NKI, and is currently not provided in the other centers. Moreover, introducing randomization in the NKI with a “no supportive care” group raises ethical concerns. ‘Therefore, this controlled observational study within different HNC centers was considered to be the most feasible design. To best approach the internal validity of a randomized study, we will adjust for case mix by using propensity score analysis [44]. Furthermore, the USC provided by the control group to HNC patients can vary among the different HNC centers. In this study, these centers are merged in one control group. Differentiation between subgroups of comparable USC will only be feasible in case sufficient number of patients is included in each of these subgroups, which will be challenging especially if one of these groups is relatively well represented in accrual numbers. To minimize the risk of selection bias we recruit sequential cohorts in all participating centers.

Another limitation of this study is the restriction of inclusion to advanced HNC patients treated with CRT or BRT. Patients treated with extensive primary surgery, such as a total laryngectomy, also have rehabilitation needs for which IHNR could be profitable. Nonetheless, we opted to select only patients treated with CRT or BRT to obtain a group as homogenous as possible. Also, as the benefits on effectiveness of interdisciplinary care compared to monodisciplinary and (in particularly) multidisciplinary care have not been proven yet, we will aim to investigate this using multiple outcome measurements. However, whether we can eventually prove these benefits is not certain. If unmet needs arise from this study, this may be relevant for improvement of rehabilitation care an incentive to also follow-up with a study including qualitative methods or implementing a HNC-specific tool such as the Patient Concerns Inventory [45]. Still, patient’s assessment of unmet needs can be difficult as patients are often not aware of the possibilities with regard to supportive care, with the result that some unmet needs remain unknown.

A phenomenon experienced in survivorship studies is the fact that awareness- and diffusion of knowledge on aspects of survivorship care, sometimes in the shape of general healthy living- and general psychosocial advice or it’s availability on the internet, makes USC a kind of moving target [46]. This leads to difficulty in establishing the exact differences between the trial arms. Finally, patients who are eligible for this study are also eligible for several other ongoing studies. If patients are included in multiple clinical studies, this may have some influence on HRQoL outcomes. Due to the multicenter nature of most of the other studies, we do not expect these studies to cause relevant differences between centers. Therefore, we believe that the impact on the estimate of effect will be negligible.

With the outcomes of this study, we aim to get more insight into the applicability and efficiency of IHNR in practice. If IHNR proves more (cost-)effective compared to USC, the availability and nationwide reimbursement through basic health insurance will contribute to a better HRQoL in this vulnerable group of patients.

## Data Availability

The dataset used and analyzed during the current study will be available from the corresponding author on reasonable request.

## Abbreviatons

ABC: Activity based costing
BRT: Bioradiotherapy
CEA: Cost-effectiveness analysis
CRT: Chemoradiotherapy
DHNS: Dutch Head and Neck Society
EORTC: European Organization for Research and Treatment of Cancer
EQ-5D-5 L: Five-level EuroQol five-dimensional questionnaire
FU: Follow-up
HNC: Head and neck cancer
HNSCC: Head and neck squamous cell carcinoma
HRQoL: Health-related quality of life
ICER: Incremental costs-effectiveness ratio
ICF: International Classification of Functioning, Disability and Health
IHNR: Interdisciplinary head and neck cancer rehabilitation
IKNL: Netherlands Comprehensive Cancer Organization - *Integraal Kankercentrum Nederland*
MCQ: Medical Consumption Questionnaire
NKI: Netherlands Cancer Institute
NZa: Dutch Healthcare Authority – *Nederlandse Zorgauthoriteit*
PREP: Preventive (swallowing) exercise program
PROM: Patient reported outcome measure
PWHHT: Dutch working group of allied healthcare in head and neck cancer *– Paramedische Werkgroep voor Hoofd-Halstumoren*
QALY: Quality-adjusted life year
QLQ-C30: Quality of life questionnaire-core 30
RTW: Return to work
UN: Unmet needs
USER-P: Dutch Utrecht Scale for Evaluation of Rehabilitation-Participation – *Utrechtse Schaal voor Evaluatie van Revalidatie-Participatie*
USC: Usual supportive care
WAI: Workability index

## References

[1] Netherlands Comprehensive Cancer Organisation. Incidence of head and neck cancer in the Netherlands. [in Dutch]. Available from: https://www.cijfersoverkanker.nl/selecties/Dataset_1/img5d19afb60a6fe. Accessed 1 July 2019.

[2] Passchier E, Stuiver MM, van der Molen L, Kerkhof SI, van den Brekel MW, Hilgers FJ (2016). Feasibility and impact of a dedicated multidisciplinary rehabilitation program on health-related quality of life in advanced head and neck cancer patients. Eur Arch Otorhinolaryngol.

[3] Giuliani M, McQuestion M, Jones J, Papadakos J, Le LW, Alkazaz N (2016). Prevalence and nature of survivorship needs in patients with head and neck cancer. Head Neck.

[4] Netherlands Comprehensive Cancer Organisation. Cancer Guideline (2011). Available from: https://www.oncoline.nl/medisch-specialistische-revalidatie-bij-oncologie. Accessed 1 July 2019.

[5] Licitra L, Keilholz U, Tahara M, Lin J-C, Chomette P, Ceruse P (2016). Evaluation of the benefit and use of multidisciplinary teams in the treatment of head and neck cancer. Oral Oncol.

[6] Clarke P, Radford K, Coffey M, Stewart M (2016). Speech and swallow rehabilitation in head and neck cancer: United Kingdom National Multidisciplinary Guidelines. J Laryngol Otol.

[7] Eadie TL (2003). The ICF: a proposed framework for comprehensive rehabilitation of individuals who use alaryngeal speech. Am J Speech-Lang Pathol.

[8] Eades M, Murphy J, Carney S, Amdouni S, Lemoignan J, Jelowicki M (2013). Effect of an interdisciplinary rehabilitation program on quality of life in patients with head and neck cancer: review of clinical experience. Head Neck.

[9] van Weert E, Hoekstra-Weebers J, Grol B, Otter R, Arendzen HJ, Postema K (2005). A multidimensional cancer rehabilitation program for cancer survivors: effectiveness on health-related quality of life. J Psychosom Res.

[10] Dieperink KB, Johansen C, Hansen S, Wagner L, KA K, Minet LR (2017). Male coping through a long-term cancer trajectory. Secondary outcomes from a RTC examining the effect of a multidisciplinary rehabilitation program (RePCa) among radiated men with prostate cancer. Acta Oncol.

[11] Leclerc AF, Foidart-Dessalle M, Tomasella M, Coucke P, Devos M, Bruyere O (2017). Multidisciplinary rehabilitation program after breast cancer: benefits on physical function, anthropometry and quality of life. Eur J Phys Rehabil Med.

[12] Khan F, Amatya B, Drummond K, Galea M (2014). Effectiveness of integrated multidisciplinary rehabilitation in primary brain cancer survivors in an Australian community cohort: a controlled clinical trial. J Rehabil Med.

[13] WHO. International Classification of Functioning, Disability and Health (ICF). Available from: http://www.who.int/classifications/icf/en/. Accessed 19 Mar 2018.

[14] Tschiesner U, Rogers S, Dietz A, Yueh B, Cieza A (2010). Development of ICF core sets for head and neck cancer. Head Neck.

[15] Netherlands Cancer Institute. HNR 2.0 (version 2006). Available from: https://www.avl.nl/flipbooks/HHR-HNR%202.0/index.html#p=1. Accessed 19 Mar 2018.

[16] Scott NW, Fayers P, Aaronson NK, Bottomley A, de Graeff A, Groenvold M (2008). EORTC QLQ-C30 reference values manual.

[17] Retèl VP, van der Molen L, Hilgers FJ, Rasch CR, l’Ortye AA, Steuten LM (2011). A cost-effectiveness analysis of a preventive exercise program for patients with advanced head and neck cancer treated with concomitant chemo-radiotherapy. BMC Cancer.

[18] Mewes JC, Steuten LM, IJzerman MJ, Van Harten WH (2012). Effectiveness of multidimensional cancer survivor rehabilitation and cost-effectiveness of cancer rehabilitation in general: a systematic review. Oncologist.

[19] Dutch Society for Otorhinolaryngology. Guideline for head and neck tumors. [in Dutch]. https://richtlijnendatabase.nl/richtlijn/hoofd-halstumoren/hoofd-halstumoren_-_korte_beschrijving.html. Accessed 1 July 2019.

[20] Giesinger JM, Kieffer JM, Fayers PM, Groenvold M, Petersen MA, Scott NW (2016). Replication and validation of higher order models demonstrated that a summary score for the EORTC QLQ-C30 is robust. J Clin Epidemiol.

[21] Aaronson NK, Ahmedzai S, Bergman B, Bullinger M, Cull A, Duez NJ (1993). The European Organization for Research and Treatment of Cancer QLQ-C30: a quality-of-life instrument for use in international clinical trials in oncology. J Natl Cancer Inst.

[22] Fayers PM, Aaronson NK, Bjordal K, Grønvold M, Curran D, Bottomley A (2001). EORTC QLQ-C30 scoring manual.

[23] Sherman AC, Simonton S, Adams DC, Vural E, Owens B, Hanna E (2000). Assessing quality of life in patients with head and neck cancer: cross-validation of the European Organization for Research and Treatment of Cancer (EORTC) quality of life head and neck module (QLQ-H&N35). Arch Otolaryngol Head Neck Surg.

[24] SZENDE AGOTA, OPPE MARK, DEVLIN NANCY (2007). EQ-5D Value Sets.

[25] Lievens Y, Van den Bogaert W, Kesteloot K (2003). Activity-based costing: a practical model for cost calculation in radiotherapy. Int J Radiat Oncol Biol Phys.

[26] Bouwmans C, Hakkaart-van Roijen L, Koopmanschap M, Krol M, Severens J, Brouwer W (2013). Manual of the iMTA medical consumption questionnaire (iMCQ).

[27] Hakkaart-Van Roijen L, Van der Linden N, Bouwmans C, Kanters T, Swan Tan SK (2015). Methodologie van kostenonderzoek en referentieprijzen voor economische evaluaties in de gezondheidszorg [manual for economic evaluations: methods and standard cost prices for economic evaluations in health care].

[28] Dutch Health Authority (NZa). DBC product finder for tariffs (2017). Available from: https://puc.overheid.nl/nza/doc/PUC_236092_22/1/. Accessed 1 July 2019.

[29] De Zwart B, Frings-Dresen M, Van Duivenbooden J (2002). Test–retest reliability of the work ability index questionnaire. Occup Med.

[30] De Boer A, Verbeek J, Spelten E, Uitterhoeve A, Ansink A, De Reijke T (2008). Work ability and return-to-work in cancer patients. Br J Cancer.

[31] Post MW, van der Zee CH, Hennink J, Schafrat CG, Visser-Meily JM, van Berlekom SB (2012). Validity of the Utrecht scale for evaluation of rehabilitation-participation. Disabil Rehabil.

[32] Ma Xuelei, Zhang Jing, Zhong Wuning, Shu Chi, Wang Fengtian, Wen Jianing, Zhou Min, Sang Yaxiong, Jiang Yu, Liu Lei (2014). The diagnostic role of a short screening tool—the distress thermometer: a meta-analysis. Supportive Care in Cancer.

[33] Carlson LE, Waller A, AJ M (2012). Screening for distress and unmet needs in patients with cancer: review and recommendations. J Clin Oncol.

[34] National Comprehensive Cancer Network. National Comprehensive Cancer Network, NCCN clinical practice guidelines in oncology: distress management. US; 2008. Available from: https://www.nccn.org/patients/resources/life_with_cancer/pdf/nccn_distress_thermometer.pdf. Accessed June 2019 10.6004/jnccn.2019.0048 PMC6907687 31590149

[35] Cohen J (1988). Statistical power analysis for the behavioral sciences.

[36] Have Thomas R. Ten, Diggle Peter J., Liang Kung-Yee, Zeger Scott L. (1995). Analysis of Longitudinal Data. Journal of the American Statistical Association.

[37] Son H, Friedmann E, Thomas SA (2012). Application of pattern mixture models to address missing data in longitudinal data analysis using SPSS. Nurs Res.

[38] Steyerberg E.W. (2009). Clinical Prediction Models.

[39] Garrison LP, Mansley EC, Abbott TA, Bresnahan BW, Hay JW, Smeeding J (2010). Good research practices for measuring drug costs in cost-effectiveness analyses: a societal perspective: the ISPOR drug cost task force report—part II. Value Health.

[40] Koopmanschap MA, Rutten FF, van Ineveld BM, Van Roijen L (1995). The friction cost method for measuring indirect costs of disease. J Health Econ.

[41] Briggs AH, Claxton K, Sculpher MJ (2006). Decision modelling for health economic evaluation: Handbooks in Health Economic E.

[42] Fenwick E, Claxton K, Sculpher M (2001). Representing uncertainty: the role of cost-effectiveness acceptability curves. Health Econ.

[43] Raad voor de Volksgezondheid en Zorg aan de minister van Volksgezondheid, Welzijn en Sport. Zinnige en duurzame zorg. Zoetermeer; 2006. https://www.raadrvs.nl/documenten/publicaties/2006/06/07/zinnige-en-duurzame-zorg. Accessed 1 July 2019.

[44] d’Agostino RB. Tutorial in biostatistics (1998). Propensity score methods for bias reduction in the comparison of a treatment to a non-randomized control group. Stat Med.

[45] Ghazali N, Kanatas A, Bekiroglu F, Scott B, Lowe D, Rogers SNJTBotRCoSoE. The Patient Concerns Inventory: a tool to uncover unmet needs in a cancer outpatient clinic. 2013;95(3):1–6.

[46] van Waart H, Stuiver MM, van Harten WH, Geleijn E, Kieffer JM, Buffart LM (2015). Effect of low-intensity physical activity and moderate-to high-intensity physical exercise during adjuvant chemotherapy on physical fitness, fatigue, and chemotherapy completion rates: results of the PACES randomized clinical trial. J Clin Oncol.

